# Lymphoid morphology, hyperviscosity and extensive immunoglobulin deposits in myeloma

**DOI:** 10.1002/jha2.569

**Published:** 2022-09-26

**Authors:** Tushar Sehgal, Nicholas Lafferty, Linda Barton, Mohammed Altohami

**Affiliations:** ^1^ Department of Laboratory Medicine All India Institute of Medical Sciences Delhi India; ^2^ Department of Haematology University Hospital Southampton NHS Foundation Trust Southampton UK; ^3^ Department of Haematology University Hospital of Leicester NHS Trust Leicester UK

1

A 64‐year male presented with epistaxis and shortness of breath. He had haemoglobin of 92 g/L with normal white cell and platelet counts, IgGλ paraprotein of 45.2 g/L and normal creatinine and calcium levels. A blood film showed macroscopically visible immunoglobulin deposits, rouleaux formation and atypical lymphoid cells (Figure 1A: 20× objective; B: 50× objective; MGG stain). Plasma exchange led to rapid improvement in symptoms. Bone marrow (Figure 1C: H&E, 40× objective; D: Giemsa, 40× objective) showed a 90% infiltrate of mononuclear cells, many with central nuclei and ‘clock‐face’ nuclear morphology but small to medium amount of cytoplasm and general lack of perinuclear halo. The flow cytometric immunophenotype of the population was CD45^–^, CD19^–^, CD38^+^, CD56 bright and CD27^–^. Immunohistochemistry showed CD3^–^, CD5^–^, CD20^–^, CD79a^–^, CD138^+^, CD38^+^, MUM1^+^, CCND1–, CD56–, BCL2–, BCL6–, CD21–, CD23–, CD10–, μ heavy chains –, γ heavy chain weak + and λ light chain restricted mononuclear infiltrate. Fluorescence in situ hybridization showed t(4;14) and gain of *CKS1B* (1q21). Whole body magnetic resonance imaging revealed a diffuse low bone marrow T1 signal but no focal bone lesions. A diagnosis of plasma cell myeloma was made, and the patient was initiated on bortezomib, thalidomide and dexamethason and went on to have an autologous stem cell transplant.

**FIGURE 1:**
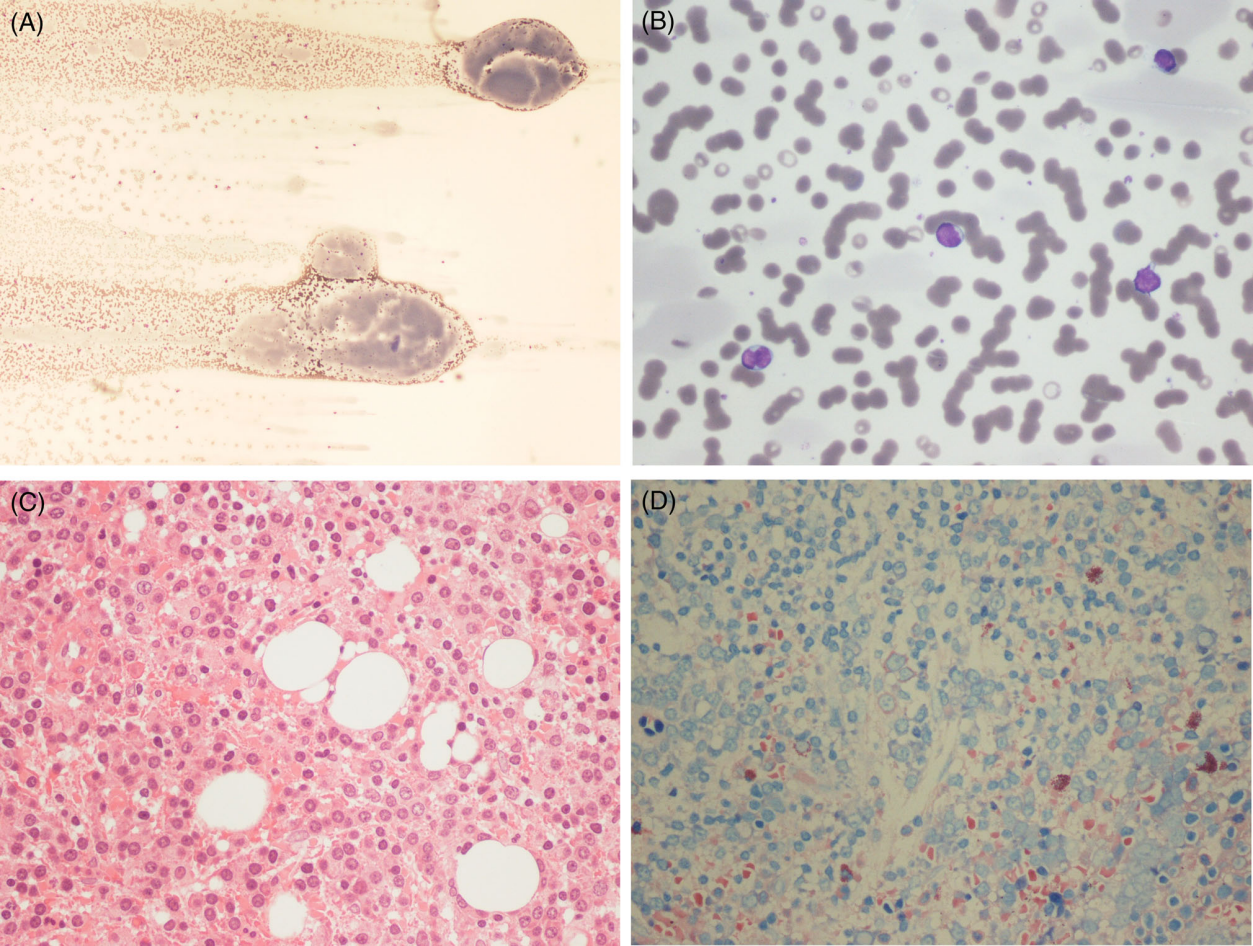
The blood film showed macroscopically visible immunoglobulin deposits, rouleaux formation and atypical lymphoid cells (A and B; MGG; 20× and 50× objective). Bone marrow biopsy showed heavy infiltration by mononuclear cells with small amount of cytoplasm (H&E and Giemsa; 400× magnification).

Differentiation of myeloma from lymphoma can be challenging in the presence of atypical clinical, morphological, immunophenotypic and/or genetic findings. Some of the shared findings are discussed below.

The classical myeloma hyper*C*alcaemia, *R*enal impairement, *A*naemia and *B*one lesions features can also be seen with lymphoma. Hyperviscosity and autoimmune phenomena are more typical of low‐grade lymphomas but are also rarely seen in myeloma. Extramedullary spread including leukaemic dissemination, lymphadenopathy and hepatosplenomegally affect 10% of myeloma patients. Histologically, the differentiation between extramedullary plasmablastic myeloma and plasmablastic lymphoma is particularly challenging.

Plasma cells in myeloma show loss of CD45, CD19 and surface immunoglobulin expression and gain of CD56, CD200, CD28 and CD117 expression. The expression of CD20, CD19 and PAX‐5 and/or absence of CD56 or CD117 is not infrequent in myeloma. Plasma cells in lymphoplasmacytic lymphoma (LPL) usually express CD45, CD19, surface immunoglobulin and PAX‐5. However, LPL cases with marked plasma cell excess, CD45 and CD19 negativity, MYD‐88 negativity and non‐IgM paraprotein have been described.

The presentation of myeloma and concomitant or metachronous lymphoma (most commonly) has been reported, including chronic lymphocytic leukaemia/small lymphocytic lymphoma (CLL/SLL) and LPL. LPL cases may show distinct plasma cell and lymphoid predominant areas.

t(4;14) has been very rarely reported in CLL and prolymphocytic leukaemia. t(11;14)(q13;q32) is seen in myeloma and in mantle cell lymphoma. Myeloma with t(11; 14) shows higher frequency of some of the classical findings of lymphoid malignancies including small lymphoid morphology, CD20 expression, oligo‐ or non‐secretary disease, IgM paraproteinaemia, surface immunoglobulin expression and leukaemic dissemination. Such cases are however always negative for MYD88 mutation.

This case demonstrates the need for integration of clinical, serological, biochemical, morphological, immunophenotypic and genetic data for accurate diagnosis of plasma cell myeloma.

## AUTHOR CONTRIBUTIONS

Mohammed Altohami and Tushar Sehgal captured the microscopic images. Nicholas Lafferty summarised laboratory data. All authors contributed to writing and finalising the manuscript.

## CONFLICT OF INTEREST

The authors declare they have no conflicts of interest.

## FUNDING INFORMATION

The authors received no specific funding for this work.

